# A search for a safer bucket to prevent children drowning at home

**DOI:** 10.5249/jivr.v9i2.805

**Published:** 2017-07

**Authors:** Alfredo Celis, María de Jesús Orozco-Valerio, Ana Cecilia Méndez-Magaña, Alfredo Celis-Orozco

**Affiliations:** ^*a*^Public Health Department, Health Campus, University of Guadalajara, Guadalajara, Jalisco, México.; ^*b*^Chemical Engineering Department, University of Guadalajara, Guadalajara, Jalisco, México.

**Keywords:** Drowning, Water storage, Infant, Child preschool, Accident prevention

## Abstract

**Background:**

Unintentional drowning is the leading cause of death for children younger than 5 years old. A bucket is one of the most common water container in which children can drown. The objective of this work was to evaluate the base diameter of a bucket and the necessary force to shed it.

**Methods:**

This was an experimental study. We used six galvanized buckets of different diameters. Each selected bucket was pulled using a pulley with other buckets full of water until the water spilled out. The statistical analysis was done by linear regression with p less than 0.05 as statistically significant.

**Results:**

This research shows a direct relation between the wide base diameter (in a bucket 23 cm high, 25 cm rim, with a 20 cm water depth) and the strength required to spill the liquid contents (β= 1.21; x= diameter of the base in centimeters; α= 14.59; r= 0.99 and p less than 0.001).

**Conclusions:**

We conclude that the bucket structure could determine the risk of child drowning. The risk could increase directly as its base width increases.

## Introduction

A drowning is a common and preventable health problem^1^ and it is one of the leading causes of lethal non-intentional injuries in children younger than 5 years old. ^2,3^ The locations of bodies of water associated with drowning vary with children’s ages and stage of devel-opment, though the “home” is one of the most fre-quently reported location. ^1,4,5^ At home, children around 3-4 years old drown most frequently in cisterns, wells, sinks or trenches, whereas children around 1 or 2 years old drown in buckets or bathtubs. ^3,4,6^ In the Guadalajara Metropolitan Area (Jalisco State, México), buckets represent the second most frequent body of water where children 1- 4 years old drown at home, representing 21.2% of all cases. In first place was the underground cistern, with 39.4%. ^7,8^ According to Leon,^8^ preschool children can drown when the water depth reaches 2.5 cm, and the risk increases as water reaches depths of 20 or more centimeters.^9^

Infant and preschool children have characteristics and behaviors that increase the risk of drowning. These in-clude imperfect motor coordination, staggering gait, fragmentary observation, incapacity, development of independence, and an interest in knowing what is around them with no difference of danger.^10^ Besides, when they fall, they often have difficulties getting up because their gravity center is close to the head and their muscle mass is not sufficient to tumble a bucket. ^11^

The preventive measures for these cases, issued by different organizations, include avoiding leaving buckets filled with water or other liquids when not in use, or proceeding with extreme caution when using buckets with water in the presence of children. These recommendations have the purpose of regulating the behavior and habits of people.^12-14^ These findings are all based on studies, but no one has examined the actual structure of buckets, except for their size (bigger buckets are riskier than smaller). However, we believe there is much to explore regarding this topic; for example, the shape of a bucket: at a market we can choose from cylindrical to truncated cone bucket shapes. It is clear that a bucket with a smaller base is less stable and is easier to floor, but no one has measured it; therefore, it could be an important factor to prevent drowning in children be-cause, to support preventive measures based on educa-tion, it is also necessary to introduce legislative and engineering changes.^15^ That is the aim of this study to measure the stability of buckets with different base/rim ratios, and the force needed to floor them when filled with water.

## Methods

**Figure 1:**
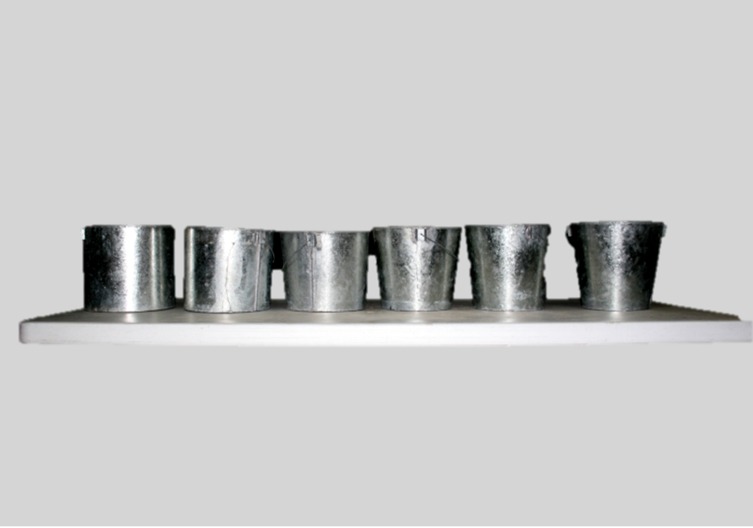
Types of buckets used.

**Figure 2:**
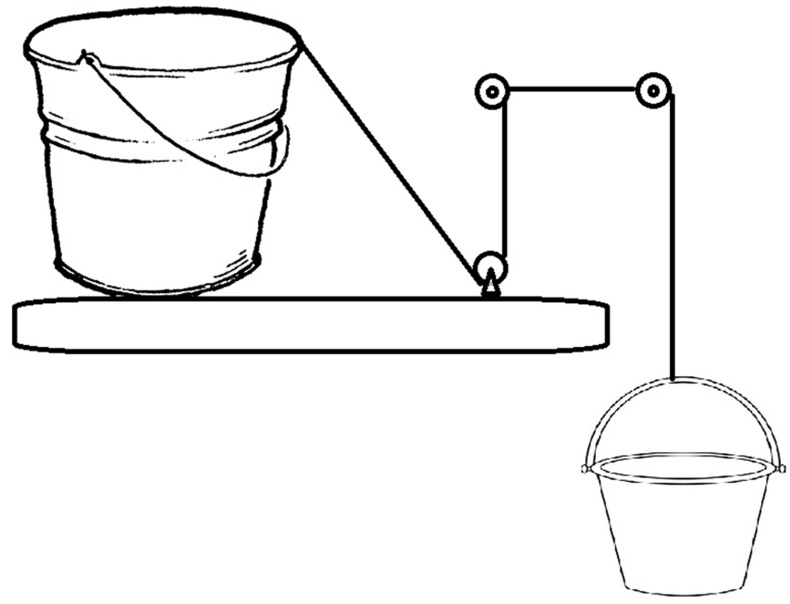
Traction movement of buckets

We designed six types of metallic buckets, all of them 23 cm high with a top rim diameter of 26 cm, but with varying base diameters: 26.00, 24.67, 23.26, 21.75, 20.14, and 18.38 cm (Figure 1). All of the buckets were filled with a 20 cm high column of water. Later, we applied progressive pressure in a 45° direction towards the floor until the buckets tilted enough to spill the water they contained. The pressure was applied via a rope fastened to the bucket rim, held and directed by three pulleys. At the other end was another bucket with liquid. The second bucket was progressively felt with water until the first bucket tilted and spilt the water (Figure 2). At that moment, we stopped filling and measured the weight of the water needed to tilt the bucket in kilo-grams using a bascule. The data recorded were ana-lyzed through a correlation graph and a simple linear regression with “diameter of the base” as the independ-ent variable and “strength in kg require to tilt” as the dependent variable.

## Results

**Figure 3:**
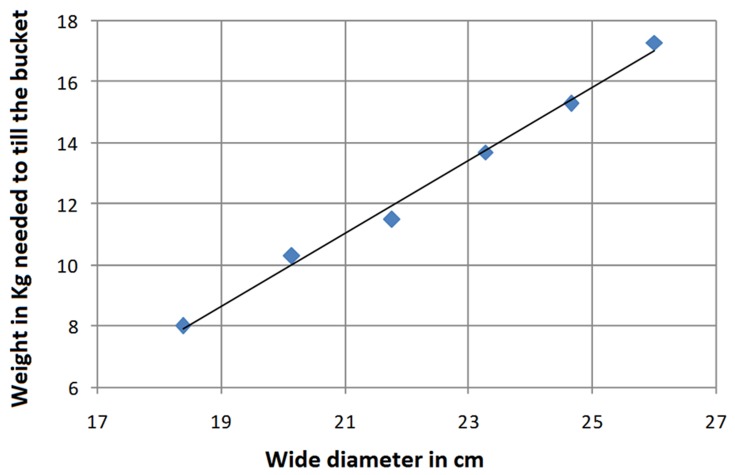
Correlation and regression graph.

The correlation graph showed a direct relation between the base diameter of the bucket and the strength re-quired to spill the liquid content (Figure 3). The regression equation showed a direct relation between “diameter of the base” and “strength in kg require to tilt”, with the following statistics: β = 1.21; x = diameter of the base in centimeters; α = 14.59; r = 0.99 and p<0.001.

## Discussion

This study shows a direct relation between the base diameter of the bucket and the strength needed to tilt it until its contents are spilled. The bucket with the widest base (26.0 cm in diameter) required 17.3 kg to tilt it, whereas the bucket with the narrowest diameter (18.38 cm) only needed 8.0 kg. Since infants of 11 months old or older weigh approximately 9.7 kg or more, if they fall into a buckets full of water, they could spill its contents due only to their weight when the base of the deposit is narrow (at least 50% of the diameter of the rim of the bucket) and the height of the bucket is 23 cm and the rim has a diameter of 26 cm. However, it is important to mention that, in our study, the force ap-plied to tilt the bucket was in a 45° direction. Since children do not usually fall into a bucket in that direction we cannot assure that all buckets with narrow bases will tilt when children fall into them, but that the chances of this happening will increase as long as the bucket base is narrower than the opening. 

This study has some limitations related to our test buckets, mainly regarding their material, size, and water capacity. Besides, our galvanized buckets are surely not a representative sample of the buckets we typically find in Mexico.

Even though this is a health problem, there is very little information about children drowning in buckets in Mexico.^16^ The death certificate includes a section to describe the drowning event; however, this is not required to describe the body of water where children drown, and neither is its container.^16^ Therefore, we do not know which kind of bucket is most frequently associated with child drowning. For our study, we used a commercial sized bucket and focused on exploring the shape of them to find the easiest design for a child to floor a bucket full of water once they have fallen into it.

We chose the height of 23 cm for our buckets because it is about the same height for the most available commercial bucket in the regional market. The water capacity of our galvanized buckets averaged 9 liters, about the same as the commercial plastic and galvanized commercial buckets available in Mexico. In the stores, we tend to see more plastic than metal buckets; nevertheless, we decided to build our test buckets with galvanized sheet because it was difficult for us to form them in plastic (galvanized metal is commonly worked in workshops in our city, while there is no factory available that offers it at a reasonable price). The weight for commercial galvanized buckets is approximately 900 g, while for plastic buckets it is approximately 300 g. Our workshop galvanized buckets are 600 g. We understand that there are important differences between these two kinds: plastic is more flexible and less heavy than metal, and the same test with the plastic ones should differ from the metal buckets. However, we did not anticipate big differences in our results, mainly because the weight difference between our tested buckets and the commercial plastic buckets is about 300 g in favor of the galvanized ones, so we worked with an intermediate weight. Thus, the height, weight, and water capacity of our tested buckets are very similar to those of commercial buckets.

In the market, there are many buckets of different sizes and shapes; however, these are not available with the base/rim ratio and height we were interested in testing. Besides, since we were interested in the force applied to tip, the contents and the weight of our tested bucket are not too different from the commercial buckets, we considered that our results would not differ from those we could obtain from using plastic ones.

The most frequently reported bucket internationally related to children drowning mortality is a 5 gallon bucket.^17,18^ This type of bucket is mainly used as a container to sell fluids (oil, paint) or powders (detergent, flour), which comes “free” with a product. After they have been used, they are often sold in informal markets as empty buckets. Their cylindrical structure is designed for stability because they are stacked. These buckets are more dangerous than our tested buckets because they are cylindrical, higher, and heavier (mainly when filled), but we do not anticipate a different behavior in relation to the base/rim ratio. If this bucket were produced with a smaller base than its rim, it would be easy to tip.

Our main purpose was to demonstrate that the shape of the bucket should matter because it can prevent drowning among toddlers, no matter the material used to make a bucket. However, our focus on the base/rim ratio leaves others issues of bucket design unaddressed, specifically regarding changes that could improve our children’s safety against drowning. For example, covering the bucket opening with a lid would prevent children from falling and drowning in buckets. In addition, a deformable bucket would increase the chances of spilling the water content.

Previous published and recommended safety measures include maintaining buckets that are empty turned upside down^13,19^ and stress supervision while buckets are filled and in use. Although we support all of these safety measures, from this study, we would choose to promote the use of buckets with a wide diameter equal to or lesser than 50% of the top rim. Other possible modifications in the bucket structure that need further study include top covers and body deformable materials.
